# Relationship between health literacy level and sexual function in women in the Northwest of Iran in 2020- a cross sectional study

**DOI:** 10.1186/s12905-023-02322-2

**Published:** 2023-04-11

**Authors:** Ameneh Barikani, Mahsa Samani kia, Atefeh Khoshkchali, Monirsadat Mirzadeh

**Affiliations:** 1 Department of Community and Preventive Medicine, Children Growth Research Center, Qazvin University of Medical Sciences, Qazvin, Iran; 2 School of Medicine, Qazvin University of Medical Sciences, Qazvin, Iran; 3 Student Research Committee, School of Health, Isfahan University of Medical Sciences, Isfahan, Iran; 4 Department of Community and Preventive Medicine, Metabolic Diseases Research Center, Research Institute for Prevention of Non-Communicable Diseases, Qazvin University of Medical Sciences, Qazvin, Iran

**Keywords:** Health literacy, Sexual function, Women, Married, Education

## Abstract

**Background and purpose:**

Sexual health means coordination and adaptation of physical, emotional, intellectual and social aspects of human beings. One of the variables that affect sexual function and sexual satisfaction is health literacy. The aim of this study was to investigate the relationship between health literacy level and sexual function in married women in Qazvin health centers.

**Methods:**

In the cross-sectional study, 340 married women were selected from four health centers in Qazvin, Iran, in 2020. These selected centers were chosen randomly from 26 health centers. Participants were included in the study by using the proportional selection method based on the sample size of the all health centers. Data collection tools include three questionnaires: demographic information, The Health Literacy Questionnaire (HELIA), and Female Sexual Function Index (FSFI). Data were analyzed using SPSS 24 software. A significance level of P < 0.05 was considered for statistical analyses.

**Results:**

The highest and lowest scores of dimension’s sexual function are satisfaction, pain, and lubricant, respectively. The level of women’s health literacy in Qazvin was inadequate and borderline (56.4%). Each of the sexual function dimensions had significant positive correlations with health literacy (P < 0.001). There was a significant relationship between health literacy level with age, education, and occupation (P < 0.05). According to linear regression analysis, with the increase in years of marriage, sexual function is decreased (P < 0.02).

**Conclusion:**

Health literacy was inadequate in more than half of the study sample and health literacy was significantly associated with sexual function. Educational programs were necessary in order to promote women’s health literacy in health centers.

## Introduction

The level of knowledge and awareness of the population towards their health status and effective factors are very important. Women have the most important role in society and family [1, 2]. Sexual health is one of the basic issues. Sexual health means the coordination and adaptation of the physical, emotional, intellectual, and social aspects of human beings [3], which leads to the promotion of personality, relationships, and love. These relationships are affected by many factors such as cognition, interpersonal relationships, knowledge and attitudes, culture, and religion [4].

Sexual disorders are prevalent in men and women and need intervention [5, 6]. Sexual disorders in women include persistent or recurrent disorders in four areas; sexual desire, sexual arousal, pain during intercourse, or the inability to reach orgasm. Social and cultural barriers, taboos, and misunderstandings make it difficult to accurately estimate the prevalence of these disorders. These disorders can affect quality of life, self-confidence, temperament, spouse relationships, and daily activities [7–9]. Sexual function is common in both sexes. The prevalence of this disorder in the United States is 43% in women and 31% in men [10]. In a study in Iran, this amount was 31.5% in women. The prevalence of this disorder increases with age [7]. About 25–63% of women suffer from sexual disorders [11–13].

Most medical treatments for sexual function are not proven [14, 15]. The impact of economic and social factors and the level of literacy and income are the most important social variables affecting health. One of the variables that affect sexual function and sexual satisfaction is health literacy. Literacy is effective in determining employment, income, and social status of individuals, but only a high level of literacy is not enough because a person may have a high level of education but not understand health issues and make decisions that are inappropriate for his health, so the issue of “health literacy” was very important. The aspects of health literacy are broad and can range from the ability to read a text to understanding the information provided by a health worker [16].

Health literacy is a person’s capacity to obtain, interpret, and understand the basic information about health services that is necessary for appropriate decision- making [17]. In other words, health literacy is a set of reading, listening, analysis, and decision-making skills and the ability to apply these skills in health situations that do not necessarily go back to years of schooling or general reading ability [18]. The prevalence of sexual function in Iranian women is different, but there have been few studies on health literacy and its relationship with sexual disorders among Iranian women [3, 7, 19–23]. In the Sahib al-Zamani et al. study on the relationship between health literacy and sexual function and satisfaction among infertile couples referred to the Royan Infertility Institute, the results showed that health literacy among most couples is borderline and that its adverse effects on sexual function and sexual satisfaction are confirmed [12]. In the Kilfoyle et al. study, health literacy plays an important role in reproductive knowledge and behavior [24]. The purpose of this study was to assess the relationship between health literacy and sexual function in married women that referred to health centers in Qazvin.

## Method

In this cross- sectional study, 340 married women were selected from four health centers in Qazvin, Iran, in 2020. These selected centers were chosen randomly from 26 health centers. Participants were included in the study by using the proportional selection method based on the sample size of all health centers in Qazvin. The sample size calculated based on the study of Sahebalzamani [11] and et al. by using the sample size formula (r correlation = 0.17), which estimated 340 individuals.

$$\begin{array}{l}n = {\left[ {\frac{{{Z_\alpha } + {Z_\beta }}}{c}} \right]^2}\\c = 0.5*Ln[(1 + r)/(1 - r)]\end{array}$$

Inclusion criteria included the following:

• Married women over 18 years old • Having active health record in health centers • Having Literate.

And the exclusion criteria included the following: • Women with a history of mental illness and medication (taking antipsychotics, SSRIs, blood pressure medications, and diabetes) • Disinclination to participate in the study.

At first, the purpose of the study was explained to the participants. A three-part questionnaire containing demographic information (age, level of education, occupation, income, number of children, occupation and education of the spouse), Health Literacy Questionnaire (HELIA) and Women’s Sexual Function Index (FSFI) were completed by participants. The only native Iranian tool is the Iranian Adult Health Literacy Questionnaire or Health Literacy for Iranian Adults (HELIA) developed by Montazeri et al. [25]. The designers of HELIA believe that one of the most important advantages of this tool is its generality, so that this tool does not belong to a class, occupation, education, age group or any other specific range and can be used for different population groups. This questionnaire has 33 items: access (6 items), reading skills (4 items), understanding (7 items), appraisal (4 items), and decision-making (12 items). Answers are also determined on a five-point Likert scale (from always to never). The scores of this questionnaire were calculated as subscale scores and total scores. The range of subscale scores is between 4 and 20 for reading, 6 to 30 for access, 7 to 35 for understanding, 4 to 20 for appraisal, and 12 and 60 for decision dimension. The early scores of the five dimensions of health literacy will be calculated and then converted into a standard score between zero and 100. According to this way of scoring, scores of zero to 50 are considered insufficient health literacy, 50.1 to 66 as borderline health literacy, scores 66.1 to 84 as adequate health literacy, and scores 84.1 and up to 100 are considered excellent health literacy [13]. The 19-item Female Sexual Function Index measures women’s sexual function in six independent areas: desire (2 questions), arousal (4 questions), moisture (4 questions), orgasm (3 questions), satisfaction (3 questions), and sexual pain (3 questions) [26], and is based on a 5-pointLikert scale [27].

The questionnaire has been used in many studies abroad and has shown a high degree of internal consistency and reliability, as well as validity [28]. Evaluation of the validity and reliability of the Iranian version of the Women’s Sexual Function Index was conducted by Mohammadi et al. in 2008 [29, 30].

## Data analysis and statistical tests

Data were entered into SPSS version 24. According to the non-parametric distribution of samples in the statistical population, parametric tests such as Mann-Whitney, Kruskal-Wallis, and Spearman correlation tests were determined. The linear regression analysis was used to predict sexual function by applying demographic variables and dimensions of health literacy. Significance level of less than 0.05. was considered.

## Results

most of the participants’ education and job were diploma (59.7%) and unemployment (73.2%), respectively. The age of 183 individuals (53.8%) was 20–40 years old, and the age of 144 individuals (42.4%) was older than 40 years old. Among participants, 112 (32.9%) individuals had more than 20 years of marriage.

According to the results, 79 individuals had inadequate literacy (23.6%), 110 (32.8%) borderline, 82 (24.5%) participants had adequate literacy, and 64 participants (19.1%) had excellent literacy. The scores of health literacy in selecting women include decision making (Median = 68.7, IQR = 54.1–85.4), understanding (Median = 66.07, IQR = 50-85.7), appraisal (Median = 62.5, IQR = 43.7–81.2), access to information (Median = 62.4, IQR = 50–79), and reading (Median = 56.2, IQR = 43.7–75) respectively. The highest scores for sexual function are satisfaction (Median = 4, IQR = 2.4–4.8) and desire (Median = 3.6, IQR = 2.4–4.8) and the lowest scores are for pain (Median = 3, IQR = 1.2–4.4) and lubrication (Median = 3, IQR = 2.4–3.6).

The results showed the significant relationship between demographic characteristics and scores of health literacy with sexual function (P < 0.001) (Table 1).

**Table 1:** relationship between HELIA and FSFI with age, years of marriage, education, and job

variables	HELIA Median (IQR)	P value	FSFI Median (IQR)	P value
**Age(year)**				
< 20 20–40 > 40	72.7(51.9–90.4) 66.6(57.6–81.5) 56.5(41.5–74.7)	< 0.001	23(19.7–28.5) 22.7(18.7–25.3) 18.9(9.2–22.9)	< 0.001
**Years of marriage**				
< 10 10–20 > 20	72.7(60.1–86.6) 65.1(53.3–75.9) 52.35(37.4–64.4)	< 0.001	24(19.8–26.3) 20.7(16.3–24.4) 17(7-22.1)	< 0.001
**Education years**				
< 12 ≥ 12	55.3(42.9–65.4) 76.8(65.1–93.5)	< 0.001	19.2(13.3–23.8) 23.2(19.5–25.9)	< 0.001
**Job**				
Employment Unemployment	77.5(66.4–94.5) 59.4(45-71.3)	< 0.001	24.2(20.9–26.3) 19.5(13.9–23.9)	< 0.001

There was a strong and positive correlation between the years of marriage (r=-0.386), age (r=-0.288) and years of education (r = 0.59) of the participants with health literacy (P < 0.001). There was a significant relationship between sexual function and the occupational status of women (P < 0.001). The highest score for sexual function was observed in employed participants (22.94). Education level had a statistically significant relationship with the sexual function score (P < 0.001). There was a significant negative correlation (P < 0.001, r = -0387; P < 0.001, r = 0344) between the years of marriage and the age of the participants with sexual disorders, respectively. It was shown that there was a significant and positive correlation between total health literacy and dimensions of sexual function (Table 2).

**Table 2:** Correlation coefficient of total health literacy score with dimensions of sexual function in women

	pain	Orgasm	Satisfaction	Arousal	lubrication	desire
r	0.18	0.29	0.345	0.34	0.25	0.29
P _value_	< 0.001	< 0.001	< 0.001	< 0.001	< 0.001	< 0.001

The results of linear regression analysis showed that each dimension of health literacy (access to information, reading, understanding, appraisal, and decision- making), age, years of marriage, and education as independent variables had a significant relationship with sexual function (P < 0.001). Access to information could lead to an increase in the chance of sexual function by 0.29, but in the multivariate model, it led to a decrease of 0.29, but it was not statistically significant (P < 0.42). In the multivariate model, only years of marriage had a significant relationship with sexual function (P < 0.02). With the addition of one year to the years of marriage, sexual function decreased by 0.25 (Table 3).

**Table 3:** Linear regression analysis for prediction of sexual function according to health literacy dimensions and demographic factors

	Unadjusted			Adjusted		
	B	Beta	P value	B	Beta	P value
Access to information	0.29	0.32	< 0.001	-0.29	0.08	0.42
Reading	0.09	0.28	< 0.001	0.009	0.02	0.76
Understanding	0.11	0.31	< 0.001	-0.002	-0.05	0.96
Appraisal	0.1	0.3	< 0.001	0.45	0.12	0.11
Decision making	0.08	0.2	< 0.001	-0.01	-0.03	0.65
Age	-0.28	-0.36	< 0.001	-0.53	-0.06	0.42
years of marriage	-0.27	-0.4	< 0.001	-0.17	-0.25	0.02*
years of education	0.6	0.26	< 0.001	0.09	0.04	0.5

## Discussion

Health literacy affects people’s behavior, how they use information and services, and their physical and mental conditions. These variables also affect sexual function and satisfaction. Good sexual performance between couples includes a regular desire to engage in sexual activity, sexual arousal and orgasm, and increased marital satisfaction [11]. Health literacy is one of the factors that can affect quality of life and marital satisfaction. Findings of this study showed that there is a significant relationship between health literacy level with age, education, and occupation. Askarian et al., Ahmadi et al., and Ghaffari et al. supported this finding [12, 13, 16].

In the present study, the percentage of health literacy levels in the range of inadequate to very high were 21.8%, 31.3%, 27.8%, and 19.1%, respectively, which shows that the level of women’s health literacy in Qazvin is inadequate. Ghanbari et al. [31] in their study among women of childbearing age concluded that 45.4% were sufficiently literate and only 24.6% were moderately literate. The results of a study of 525 adults in Isfahan showed that 46.5% had adequate health literacy, and the average and inadequate literacy ratios were 38.0 and 15.5%, respectively [22]. Another study [27] in the UK found that 28.5% of the adult population was inadequately health literacy. Another study of African-American adults found that 65% of participants had low or inadequate levels of health literacy [32]. Due to the dependence of health literacy on social, economic, and cultural conditions, the difference between our results and other studies conducted in different regions was inevitable. In the study by Askarian et al., 57% of people and in the study by Ghaffari et al., 22. 65% of people were illiterate [12, 16]. In the Ahmadi et al. [13] study, 45.1% of the population were sufficiently literate, which contradicted the results of this study.

One of the goals of health centers could be to improve women’s access to health-related questions, such as through easier access to physicians and employees of the health sector, or by increasing public awareness of the existence of credible sources such as brochures, IVRs, and publications and encouraging them to replace these sources with unreliable ones. Among the types of sexual function in this study, the highest score is related to sexual satisfaction, and the lowest is related to lubrication and pain. In Rosen^’^s study, desire and arousal functions were the most prevalent sexual functions [33]. Desire and orgasmic function were the most and least function in the Beigi et al. study [34], but lubrication function was prevalent in the shokrollahi study [35]. Also, Molkara et al. [8] concluded that 20% of participants had sexual satisfaction with their spouse. These studies were contrary to our result because the median age of their participants was older and their measurement tools were different from ours, possibly this differences were due to racial, ethnic, and cultural differences that affect people’s expectations. The results of Grazyna and her colleagues study were similar with this study. They reported that satisfaction had the highest mean among the FSFI dimensions [36].

Based on the results of linear regression, a significant relationship was observed between years of marriage and sexual function after adjusting other variables. In this study, the increase in the number of years of marriage has led to sexual dysfunction. Contrary to these results, the study of Dehghankar et al. showed that with the increase in the years of marriage, the level of sexual satisfaction increased [37]. The difference between studies can be related to the type questionnaire used. In the study of Ramezani Tehrani et al. among women in 4 cities, the results were consistent with the present study, and the level of sexual satisfaction of women decreased with the increase in the number of years of marriage [38].

In this study, a significant relationship was observed between health literacy and sexual function. This relation was confirmed in Moghadam and Sahebalzamani reports. They found that health literacy affects attitudes, mental status, health-related behaviors, and consequently physical health status. In addition, health literacy can affect the use of information and sexual function in couples and thus have a positive effect on quality of life [11, 39].

In this regard, it can be said that health literacy can affect couples’ moods and attitudes toward sex and marriage, and, therefore, can affect their sexual satisfaction and increase the probability of pregnancy [40, 41].

Although this research has achieved its goals, there are inevitable limitations. First, this cross-sectional study was performed in only 4 health centers. Second, the variables under study depend on the socio-cultural environment. Therefore, to generalize the results to large groups in different areas, it is recommended that studies be conducted in other locations with larger samples. Also, meta-analysis of existing data can be done to better understand this field.

## Conclusion

It was concluded from this study that health literacy was inadequate in more than half of the study population and that health literacy was significantly associated with sexual function. In order to promote women’s health literacy, programs can be designed, implemented, and evaluated. Health literacy education must be one of the goals of the health centers. It is suggested that simple and educational materials be designed and used for women with average health literacy.

## Data Availability

All data generated and analyzed during this study are included in this article.

## Abbreviations

HELIA: Health Literacy Questionnaire
FSFI: Female Sexual Function Index
